# Empagliflozin inhibits the apoptosis of pancreatic β-cells through amelioration of endoplasmic reticulum (ER) stress

**DOI:** 10.1016/j.metop.2026.100488

**Published:** 2026-08-02

**Authors:** Narjes Nasiri-Ansari, Manpal S. Randeva, Christina-Maria Flessa, Ioannis Kyrou, Panagiotis Lembessis, Hippokratis Kiaris, Maria-Eleni Chondrogianni, Maria Dalamaga, Athanasios G. Papavassiliou, Harpal S. Randeva, Eva Kassi

**Affiliations:** a Department of Biological Chemistry, School of Medicine, National and Kapodistrian University of Athens, Athens, 11527, Greece; b Warwickshire Institute for the Study of Diabetes, Endocrinology and Metabolism (WISDEM), University Hospitals Coventry and Warwickshire NHS Trust, Coventry, CV2 2DX, UK; c Institute for Cardiometabolic Medicine, University Hospitals Coventry and Warwickshire NHS Trust, Coventry, CV2 2DX, UK; d Discoveries in Life Sciences Research Centre, Coventry University, Coventry, CV1 5FB, UK; e Aston Medical School, College of Health and Life Sciences, Aston University, Birmingham, B4 7ET, UK; f College of Health, Psychology and Social Care, University of Derby, Derby, DE22 IGB, UK; g Department of Physiology, School of Medicine, National and Kapodistrian University of Athens, Athens, 11527, Greece; h Department of Drug Discovery and Biomedical Sciences, College of Pharmacy, University of South Carolina, Columbia, SC, 29208, United States; i Endocrine Unit, First Department of Propaedeutic Internal Medicine, Laiko Hospital, National and Kapodistrian University of Athens, Athens, 11527, Greece; j Warwick Medical School, University of Warwick, Coventry, CV4 7AL, UK

**Keywords:** Pancreatic β-cells, Apoptosis, Necrosis, Endoplasmic reticulum (ER), ER stress, Empagliflozin, SGLT-2i

## Abstract

**Background:**

Progressive loss of pancreatic β-cell function in type 2 diabetes mellitus (T2DM) is linked to endoplasmic reticulum (ER) stress–induced apoptosis. Sodium-glucose cotransporter-2 inhibitors (SGLT-2i) have glucose-lowering and potential cytoprotective effects. We investigated whether empagliflozin protects β-cells from ER stress–mediated apoptosis and the underlying pathway.

**Methods:**

Mouse Beta-TC-6 (BTC-6) and hamster HIT-T15 pancreatic β-cell lines were exposed to tunicamycin (5 or 10 μg/mL) to induce ER stress, with or without empagliflozin (10^−8^, 10^−9^, 10^−10^ M). *SGLT-1* and *SGLT-*2 mRNA were assessed by RT-qPCR. Expression of ER stress markers (*GRP-94*, *BiP*, *PERK*, *eIF-2α*, *IRE-1*, *ATF-4*, *CHOP*) was measured by RT-qPCR; eIF-2α, phospho-eIF-2α and CHOP protein levels were analyzed by Western blot. Cell proliferation and apoptosis were quantified by XTT and Annexin V-FITC assays.

**Results:**

BTC-6 cells expressed *SGLT-1* but not *SGLT-2*. Empagliflozin (10^−8^ and 10^−9^ M) increased BTC-6 proliferation (p < 0.01 and p < 0.05 respectively). Tunicamycin caused significant apoptosis after 48h (p < 0.001). Co-treatment with empagliflozin (10^−8^, 10^−9^ M) significantly reduced tunicamycin-induced apoptosis, especially at 5 μg/ml tunicamycin (p < 0.01). Empagliflozin co-incubation lowered *PERK*, *eIF-2α*, *IRE-1α* and *CHOP* mRNA levels versus tunicamycin alone (all p < 0.05), and decreased phospho-eIF-2α (p < 0.01) and CHOP (p < 0.05) protein levels. Similar anti-apoptotic effects were observed in HIT-T15 cells.

**Conclusions:**

Empagliflozin enhances β-cell proliferation and attenuates ER stress–induced apoptosis *in vitro*, primarily via downregulation of the PERK–eIF-2α–CHOP pathway. These findings support further investigation of SGLT-2 inhibitors for preservation of β-cell survival and function.

## 1 Introduction

Type 2 diabetes mellitus (T2DM) is characterized by chronic hyperglycemia, which negatively impacts on insulin synthesis, secretion, sensitivity, and cell viability through multiple mechanisms. Underlying mechanisms include the gradual loss of insulin gene expression, changes in β-cell mass and function, chronic endoplasmic reticulum (ER) stress, oxidative stress, changes in mitochondrial number, increased inflammation, calcium homeostasis disruption, and increased lipotoxicity [[1], [2], [3]].

Pancreatic β-cells are responsible for insulin biosynthesis and secretion to maintain the glycemic homeostasis. During chronic exposure to hyperglycemia, due to peripheral insulin resistance and β-cell compensatory hypersecretion (at least initially), these cells experience substantial intrinsic and extrinsic endoplasmic reticulum (ER) stress. This stress impairs insulin biosynthesis and secretion, leading to β-cell failure and clinical onset of T2DM [4,5].

ER stress is modulated by the activation of the unfolded protein response (UPR). The UPR is a cellular reaction to ER stress that triggers the activation of three ER stress arms located in the ER membrane, namely: protein kinase RNA-like ER kinase (PERK), inositol-requiring kinase 1 (IRE1α), and activating transcription factor 6 (ATF6) [[6], [7], [8], [9], [10]]. Activation of these signaling cascades can lead to protein synthesis suppression or the promotion of various UPR target genes, including glucose-regulated protein GRP78 (also known as Bip) [11,12]. Upon autophosphorylation, the PERK pathway activates and phosphorylates the α subunit of the eukaryotic translation initiation factor 2 (EIF-2α), resulting in increased expression of the major pro-apoptotic transcription factor CHOP (also called GADD153), which causes β-cell apoptosis through the activation of apoptotic pathways. ER stress can also trigger cellular apoptosis via the activation of caspases or cooperation with proapoptotic Bcl‐2 family members, stimulating mitochondrial‐dependent cellular death [6,13].

Anti-diabetic drugs, including sodium-glucose co-transporter-2 inhibitors (SGLT-2is), have been shown to protect other organs such as the kidney, liver, and heart, partially by reducing hyperglycemia-induced ER stress and apoptosis. SGLT2is are widely prescribed insulin-independent anti-hyperglycemic agents which work independently of β-cells by inhibiting glucose reabsorption in the kidney, leading to the excretion of glucose in the urine and protecting cells against glucose toxicity [14,15].

Our group has previously demonstrated that empagliflozin, a key SGLT-2i, reduces hepatic cell apoptosis in high-fat diet (HFD)-fed mice through the reduction of HFD-induced ER stress [16]. However, it remains unclear whether SGLT2is impact on β-cell function and apoptosis by regulating the UPR. Recent evidence suggests that empagliflozin increases the β-cell mass and proliferation in streptozotocin-induced type 1 diabetes (T1D) mice [17]. Moreover, early combination therapy of empagliflozin and linagliptin increased β-cell mass, proliferation and function in *db/db* mice [18]. The beneficial effects of Iuseogliflozin, another SGLT2i, on β-cell function and mass at an early stage of T2DM have been shown in *db/db* mice with obesity and T2DM. Luseogliflozin increases β-cell mass by reducing β-cell apoptosis and enhancing β-cell proliferation [19]. Despite this evidence, little is known about the mediating mechanisms through which the SGLT2is exert their effects on the pancreatic cell apoptosis.

To explore whether empagliflozin has a direct effect on ER stress-induced β-cell apoptosis, we incubated pancreatic β/islet cell lines with various concentrations of empagliflozin in the presence of tunicamycin, a known ER stress inducer.

## 2 Materials and methods

### 2.1 Cell culture

Mouse Beta-TC-6 (BTC-6) and hamster HIT-T15 pancreatic β-cell lines were maintained in RPMI media, supplemented with 15% FBS. Cell culture was maintained at 37°C in a humidified atmosphere containing 5% CO_2_. Both cell lines were seeded in 12 well plates and serum starved 16h prior to treatment. After starvation, cells were incubated with various concentrations of tunicamycin (5 μg/ml, 10 μg/ml, 20 μg/ml) or empagliflozin (10^−8^,10^−9^ and 10^−10^ M) alone or pretreated for 1h with tunicamycin and then co-incubated with empagliflozin for 24 and 48h. Empagliflozin concentrations (10^−10^, 10^−9^, and 10^−8^ M) were selected to represent the low nanomolar range, based on clinically relevant pharmacokinetic data. In more detail, these concentrations were chosen to approximate biologically relevant free drug exposure while minimizing nonspecific effects that may occur at higher *in vitro* concentrations [20,21].

### 2.2 Cell viability assay

BTC-6 cells (10^4^ cells/well) were seeded into 96-well tissue culture plates. After 24h, cells underwent treatment with different concentrations of tunicamycin and empagliflozin alone or in combination for 24 or 48h. To assess cell viability, the XTT reagent was added to each well (XTT Cell Proliferation Assay Kit, Cayman) and cells were incubated for 2h at 37°C. The absorbance (A) of each well was then measured with a microtitre plate (ELISA) reader at a wavelength of 450 nm and the reference wavelength was 650 nm. Untreated cells served as control, and the cell viability rate was calculated as Atreatment/Acontrol × 100%.

### 2.3 Annexin V-FITC/PI-staining and flow cytometry

BTC-6 and HIT-T15 cells (10^6^) were stained using the FITC Annexin V/Dead Cell Apoptosis Kit (Trevigen 4830-01-k) according to the manufacturer's instructions. Stained cells were diluted in Annexin V-binding buffer and then the suspended cells were used to perform flow cytometry. Annexin V-FITC/PI-stained cells were analyzed using a BD FACS Calibur flow cytometer (BD Biosciences, Heidelberg, Germany). In total 10,000 cells were analyzed per measurement. The data were analyzed immediately using a BD FACS CANTO II flow cytometer.

### 2.4 RT-PCR

Quantitative real-time polymerase chain reaction (qRT-PCR) was performed as previously described [22]. Briefly, total RNA was isolated from both cell lines using NucleoSpin® RNA Plus (Macherey-Nagel, Düren, Germany). Quality of extracted mRNA was evaluated by nanodrop [16,23]. One thousand nanograms of RNA were reverse transcribed using LunaScript™ RT SuperMix Kit (New England Biolabs, Ipswich, MA, USA) in accordance with the manufacturer's instructions. The expression of *SGLT-1* and *SGLT-2* were measured in both cell lines, while the expression of *GRP-94*, *BIP*, *PERK*, *EIF-2α*, *IRE-1*, *CHOP* and *18s* were evaluated in BTC-6 cells, using SYBR Green-based qPCR Master Mix (New England Biolabs, Ipswich, MA, USA) by a qRT-PCR protocol on a CFX96 (Bio-RAD, Hercules, CA, USA). The 2^−ΔΔCT^ method was used to determine the expression level. Differentially expressed genes were identified through fold change filtering where a minimum of ≥2-fold change was considered significant. Primer sequences are listed in Table 1.

**Table 1:** List of primer sequences used for RT-PCR analysis in this study.

Gene	Forward primer	Reverse primer
*18S*	5′-GTTCCGACCATAAACGATGCC-3′	5′-TGGTGGTGCCTTCCGTCAAT-3′
*CHOP*	5′-CCACCACACCTGAAAGCAGAA-3′	5′-GGTGCCCCCAATTTCATCT-3′
*BIP*	5′-ACATGGACCTGTTCCGCTCTA-3′	5′-TGGCTCCTTGCCATTGAAGA-3′
*GRP94*	5′-TTGTGTCCAATTCAAGGTAATCA-3′	5′-TTGCTGACCCAAGAGGAAAC-3′
*EIF-2a*	5′-CAACGTGGCAGCCTTACA-3′	5′-TTTCATGTCATAAAGTTGTAGGTTAGG-3′
*ATF4*	5′-AGCCCCACAACATGAC-3′	5′-CCACCTCCAGATAGTCAT-3′
*IRE1*	5′-CTGTGGTCAAGATGGACTGG-3′	5′-GAAGCGGGAAGTGAAGTAGC-3′
*PERK*	5′-CGGAAGGAGTCTGAAACTCAGTG-3′	5′-ACTGAATTGGCTCAAAATCTGTTAG-3′
*XBP1*	5′-ACATCTTCCCATGGACTCTG-3′	5′-TAGGTCCTTCTGGGTAGACC-3′
Mouse-*SGLT-1*	5′- AAGATCCGGAAGAAGGCATC -3′	5′- CAATCAGCACGAGGATGAAC -3′
Mouse-*SGLT-2*	5′-GCAACATCGGCAGCGGTCAT-3′	5′-GCGGAGGTACTGAGGCATTGTG-3′
Ham- *SGLT-1*	5′- AAGATCCGGAAGAGAGCATC -3′	5′- CAATCAGCACAAGGATGAAC -3′
Ham- *SGLT-2*	5′-GCAACATTGGCAGCGGTCAT-3′	5′-GCGTAGGTACTGAGGCATTGTA-3′

### 2.5 SDS-PAGE and western-blot analysis

Western blot analysis was performed as previously described [23]. Briefly, whole-cell lysates were prepared in lysis buffer (Cell Signaling Technology, MA, USA). Samples containing 30 μg of protein were resolved by electrophoresis gels and transferred to a nitrocellulose membrane. After blocking for 1h with 5% skim milk in PBST, membranes were incubated overnight at 4°C with antibodies against p-EIF-2α (#9721 Cell Signaling), CHOP (#sc-166682 Santa Cruz Biotechnology) and anti-β-actin (Millipore Corporation, Billerica, MA, USA) primary antibodies. Membranes were then probed with goat anti-mouse IgG-HRP (31430, Thermo Scientific) or with goat anti-rabbit IgG-HRP conjugate (12–348, Millipore) secondary antibodies at room temperature (RT) for 1h. Detection of the immunoreactive bands was performed using the Clarity Western ECL Substrate (BioRad). β-actin served as a loading control. Densitometric analysis was performed using Image J.

### 2.6 Statistical analysis

Statistical analyses were performed using GraphPad Prism (version 7). Data are presented as mean ± standard deviation (SD). Student's paired two-tailed *t*-test was used to compare the means of quantitative variables between two matched groups. qRT-PCR data were analyzed using the non-parametric Wilcoxon signed-rank test because of the limited sample size, which precluded reliable assessment of data normality. A p value < 0.05 was considered statistically significant.

## 3 Results

### 3.1 Co-incubation of BTC-6 cells with empagliflozin reversed tunicamycin-induced decrease in cell viability

**Fig. 1:**
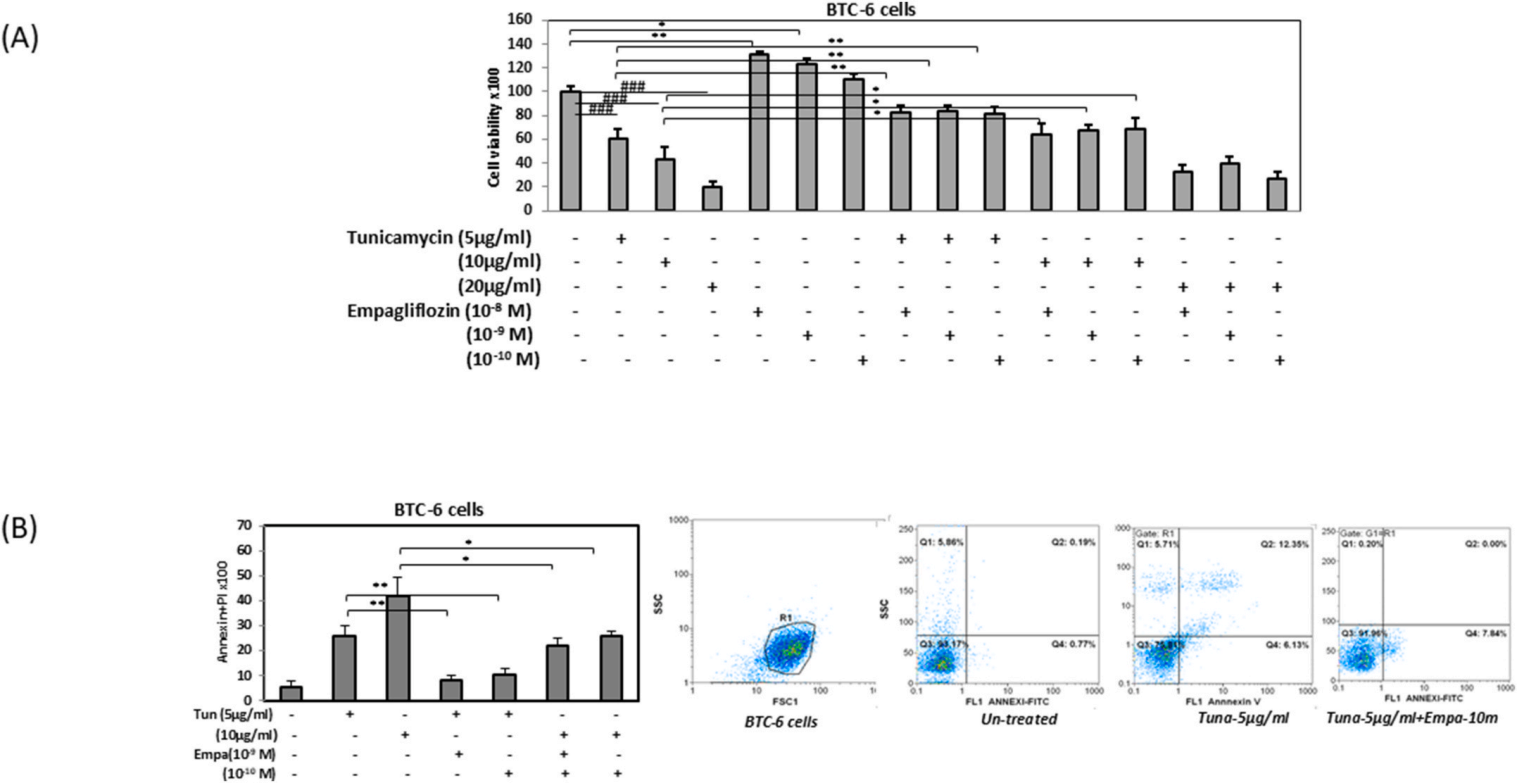
Effect of empagliflozin on tunicamycin-induced cell death in BTC-6 cells. **(A)** Cell viability of BTC-6 cells treated with tunicamycin (5, 10, or 20 μg/mL) alone or pre-incubated with tunicamycin for 1 h followed by co-incubation with empagliflozin (10^−8^, 10^−9^, or 10^−10^ M) for 48h. Cell viability was determined using the MTT assay. **(B)** Representative Annexin V/propidium iodide staining and quantification of apoptotic and necrotic BTC-6 cells following treatment with tunicamycin alone or in combination with empagliflozin. Data are presented as mean ± SD from at least three independent experiments, each including a minimum of two biological replicates. *p < 0.05, **p < 0.01 versus the untreated control; #p < 0.05, ##p < 0.01, ###p < 0.001 versus the tunicamycin-treated group.

Incubation of mouse Beta-TC-6 (BTC-6) pancreatic *β-*cells with empagliflozin (10^−8^, 10^−9^ M) resulted in significantly increased cell proliferation compared to untreated cells (p < 0.01 and p < 0.05, respectively). On the other hand, incubation of BTC-6 cells with tunicamycin for 48h reduced significantly their viability at all tested concentrations (p < 0.001). Co-incubation of cells with empagliflozin (10^−8^, 10^−9^ and 10^−10^ M) significantly inhibited tunicamycin-induced cell apoptosis with a more robust effect observed in BTC-6 cells incubated with 5 μg/ml tunicamycin (p < 0.01) compared with cells co-incubated with 10 μg/ml tunicamycin and empagliflozin (10^−8^, 10^−9^, 10^−10^ M) (p < 0.05). Of note, empagliflozin exerts no significant protective effect on the highest concentration of 20 μg/ml tunicamycin (Fig. 1A).

### 3.2 Co-incubation of BTC-6 cells with empagliflozin reversed tunicamycin-induced cell apoptosis

BTC-6 cells were incubated with tunicamycin at concentrations of 5 and 10 μg/ml alone or co-incubated with different concentrations of empagliflozin (10^−9^ and 10^−10^ M) for 48h.

Exposure of BTC-6 cells to various concentrations of tunicamycin increased significantly the percentage of apoptotic cells as compared to untreated cells. As shown in Fig. 1B, the percentage of Annexin V-positive cells, which are considered apoptotic cells, was significantly increased approximately by 25% and 41% after incubation with 5 and 10 μg/ml of tunicamycin, respectively. Consistently, the number of Annexin V-positive cells were reduced significantly after co-incubation of cells with tunicamycin (5 and 10 μg/ml) and empagliflozin at 10^−9^ and 10^−10^ M, as compared to cells incubated with the ER-inducer alone. Indeed, the co-incubation of BTC-6 cells with tunicamycin at 5 μg/ml and empagliflozin at 10^−9^ and 10^−10^ M reduced the percentage of apoptotic and necrotic cells by 68% and 59%, respectively (p < 0.01), while co-incubation of BTC-6 cells with tunicamycin at 10 μg/ml and empagliflozin at 10^−9^ and 10^−10^ M reversed cell viability by approximately 47% and 38%, respectively (p < 0.05) (Fig. 1B).

### 3.3 Co-incubation of BTC-6 cells with empagliflozin reduced the mRNA levels of ER-stress markers

First, we examined whether BTC-6 and HIT-T15 cells express *SGLT-1* or *SGLT-2* at the mRNA level. Interestingly, *SGLT-*1 mRNA was detected only in BTC-6 cells at low levels, while *SGLT-*2 mRNA was not detected in either cell line (data not shown).

**Fig. 2:**
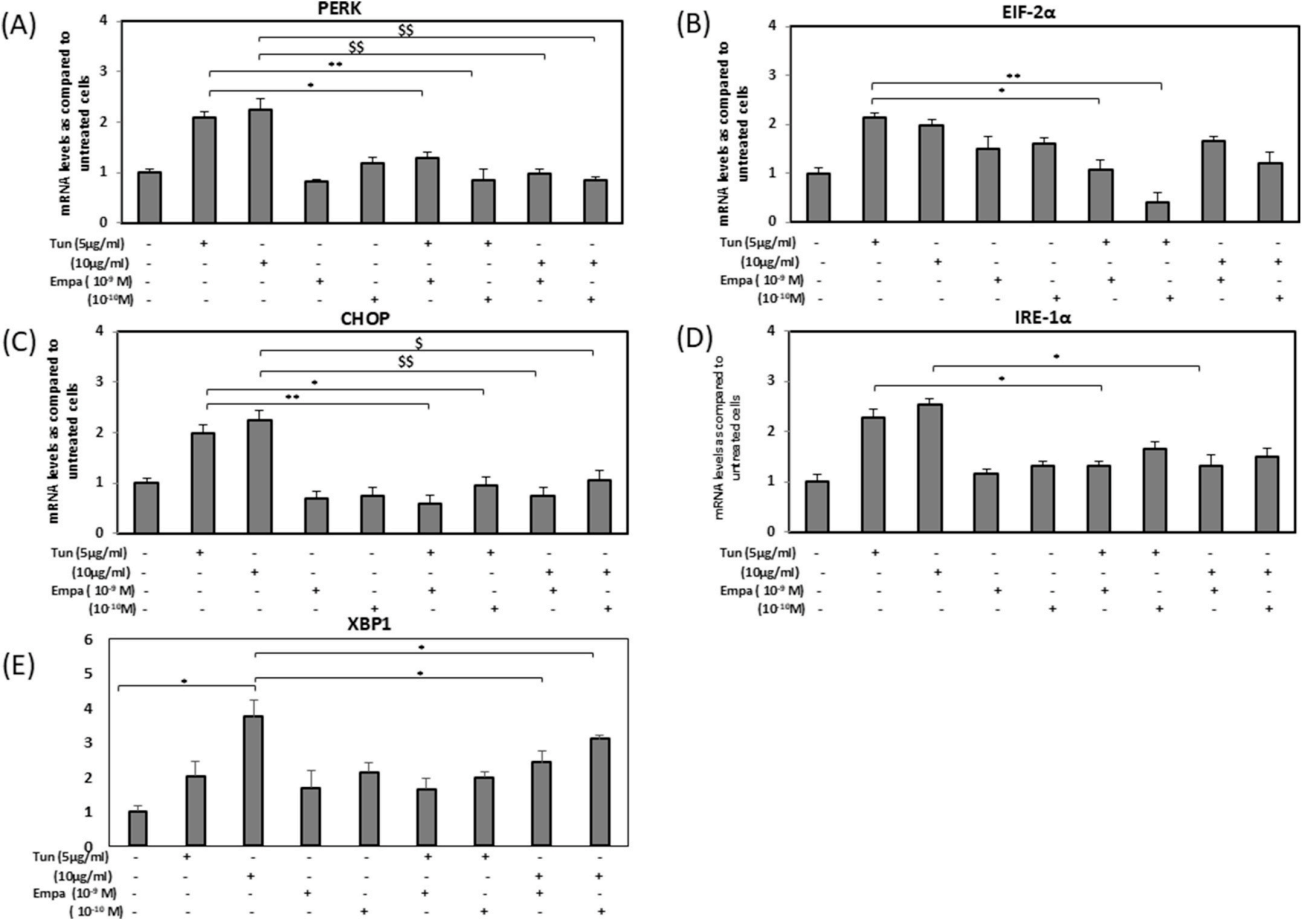
Effect of empagliflozin on the mRNA expression of endoplasmic reticulum (ER) stress–related genes in BTC-6 cells. mRNA expression levels of **(A)** PERK, **(B)** eIF2α, **(C)** CHOP, **(D)** IRE1α and **(E)** XBP1 were determined by qRT-PCR in BTC-6 cells treated with tunicamycin (5 or 10 μg/mL) alone or pre-incubated with tunicamycin for1h followed by co-incubation with empagliflozin (10^−9^ or 10^−10^ M) for 48h. Gene expression was normalized to the appropriate housekeeping gene (*18S*) and expressed relative to the untreated control. Data are presented as mean ± SD from at least three independent experiments, each including a minimum of two biological replicates. *p < 0.05 and **p < 0.01 versus the untreated control; $p < 0.05 and $$p < 0.01 versus the corresponding tunicamycin-treated group.

We next evaluated the effects of co-incubation of cells with empagliflozin on the expression and activation of ER stress markers in BTC-6 cells. Real-Time qPCR analysis revealed that the co-incubation of BTC-6 cells with tunicamycin and empagliflozin reduced ER stress-induced apoptosis through down regulation of *PERK*, *EIF-2α*, *IRE-1α*, *CHOP* and *XBP1* mRNA expression (Fig. 2). The co-incubation of cells with 5 μg/ml tunicamycin and 10^−9^ M empagliflozin significantly reduced the expression of *PERK*, *EIF-2α* and *IRE-1α* (p < 0.05) (Fig. 2A, B, D). Moreover, the expression of *EIF-2α* was also significantly reduced in cells treated with 5 μg/ml of tunicamycin and 10^−10^ M of empagliflozin (p < 0.01) (Fig. 2B).

Additionally, co-incubation of cells with higher concentration of tunicamycin (10 μg/ml) and both concentrations of empagliflozin significantly reduced the expression of *PERK* (p < 0.01), and *IRE-1α* (p < 0.05) (Fig. 2A–D). Furthermore, *XBP1* expression was significantly increased after incubation with 10 μg/ml tunicamycin and significantly diminished when cells were co-incubated with 10^−8^ and 10^−9^ M empagliflozin (p < 0.05) (Fig. 2E). The expression of *CHOP* was significantly increased (p < 0.05) in cells incubated with tunicamycin (5 μg/ml and 10 μg/ml), and was reduced significantly when cells were co-incubated with both concentrations of empagliflozin (10^−9^ and 10^−10^ M; p < 0.01 and p < 0.05, respectively) (Fig. 2C). No significant changes in *BIP*, *GRP94* and *ATF-*4 mRNA levels were observed in either condition (data not shown).

### 3.4 Co-incubation of BTC-6 cells with empagliflozin reduced the EIF-2α phosphorylation and CHOP protein levels

**Fig. 3:**
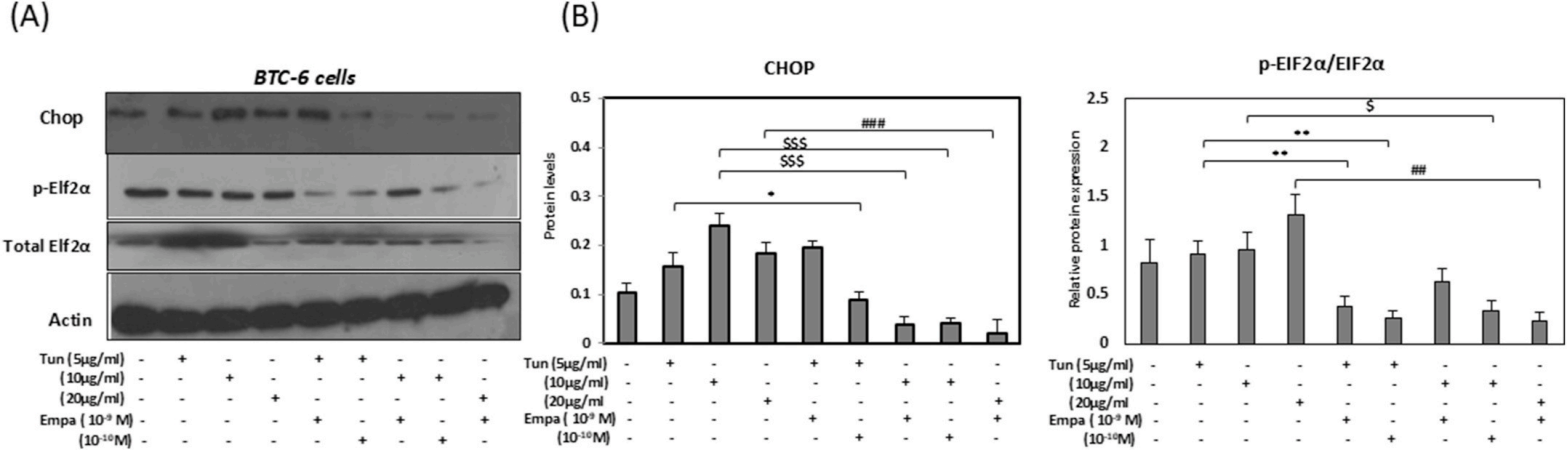
Effect of empagliflozin on CHOP and phosphorylated eIF2α/total eIF2α (p-eIF2α/eIF2α) protein levels in tunicamycin-treated BTC-6 cells. Protein expression of CHOP and phosphorylated eIF2α/total eIF2α (p-eIF2α/eIF2α) was determined by Western blot analysis in BTC-6 cells treated with tunicamycin (5, 10, or 20 μg/mL) alone or pre-incubated with tunicamycin for 1h followed by co-incubation with empagliflozin (10^−9^ or 10^−10^ M) for 48h. Representative immunoblots are shown on the left (A.), and densitometric quantification is shown on the right (B.). Experiments were performed in duplicate and repeated in two independent experiments at least. Data are presented as mean ± SD. *p < 0.05 and **p < 0.01 versus the untreated control; $$p < 0.01 and $$$p < 0.001 or ###p < 0.001 versus the corresponding tunicamycin-treated group.

Exposure of the BTC-6 cells to various concentrations of tunicamycin (5 μg/ml and 10 μg/ml) for 48h led to an increase in CHOP (p < 0.05 and p < 0.01 respectively) as well as phosph-EIF2α/EIF2α protein levels () (Fig. 3, Supplementary Fig. 1). Moreover, the co-incubation of BTC-6 cells with tunicamycin (5 μg/ml) and empagliflozin (10^−9^, 10^−10^ M) decreased the ratio of phosphorylated EIF2α/total EIF2α (p < 0.01) while 10^−10^ M empagliflozin led to significantly reduced CHOP protein levels (p < 0.05) compared to cells incubated only with 5 μg/ml tunicamycin.

Co-incubation of BTC-6 cells with 10 μg/ml tunicamycin and empagliflozin (10^−9^, 10^−10^ M) decreased CHOP protein levels (p < 0.001) while 10^−10^ M empagliflozin led to significantly diminished phosphorylated EIF2α/total EIF2α ratio (p < 0.05) compared to cells incubated only with tunicamycin. Finally, exposure of BTC-6 cells to 20 μg/ml tunicamycin and 10^−9^ M empagliflozin lowered CHOP as well as phosph-EIF2α/EIF2α protein levels (p < 0.001 and p < 0.01 respectively (Fig. 3, Supplementary Fig. 1).

### 3.5 Confirming the anti-apoptotic effect of empagliflozin using the HIT-T15 cell line

In order to verify the effect of empagliflozin on ER-stress-induced cell apoptosis in pancreatic cells, another β-cell line, namely HIT-T15 cells, was used.

**Fig. 4:**
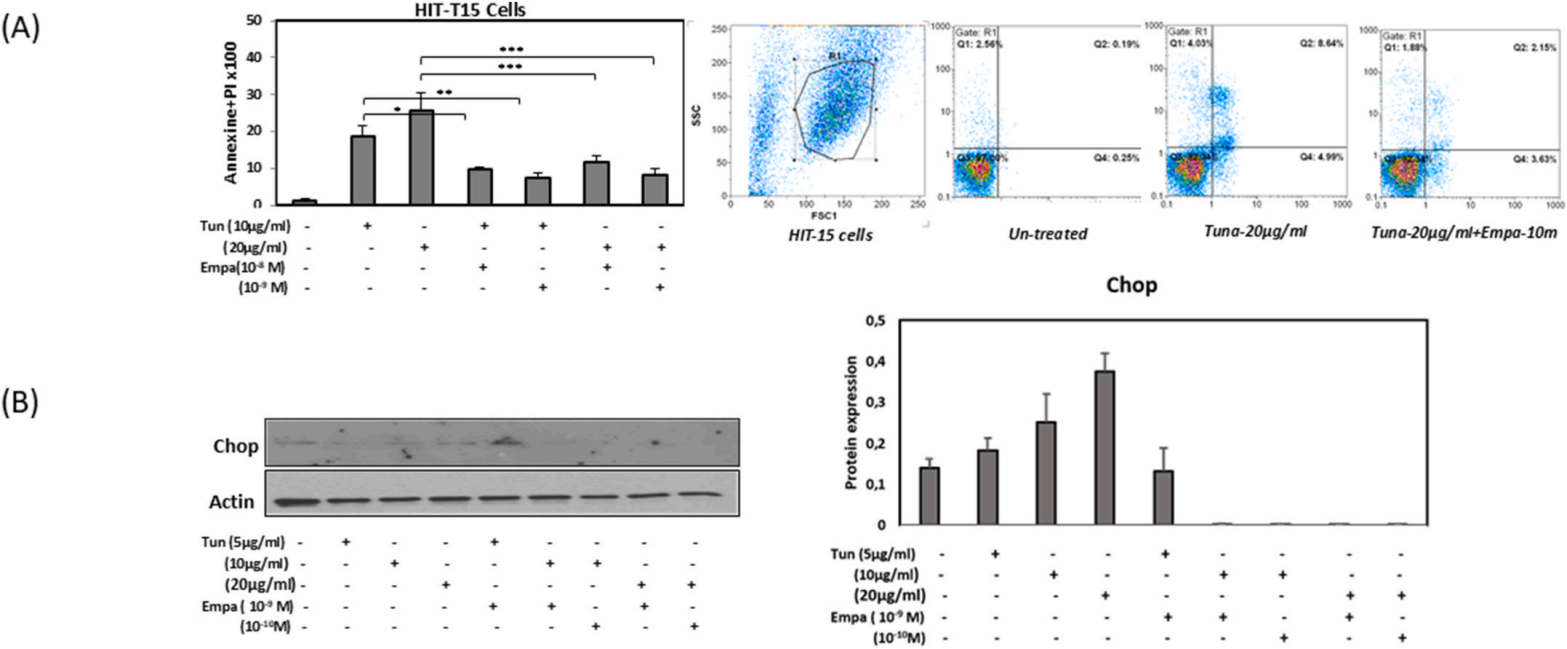
Effect of empagliflozin on apoptosis and CHOP protein expression in tunicamycin-treated HIT-T15 cells. (A) Representative Annexin V/propidium iodide (PI) flow cytometry analysis and quantification of apoptotic and necrotic HIT-T15 cells following treatment with tunicamycin (10 or 20 μg/mL) alone or in combination with empagliflozin (10^−8^ or 10^−9^ M). (B) CHOP protein expression in HIT-T15 cells treated with tunicamycin (5, 10, or 20 μg/mL) alone or pre-incubated with tunicamycin for 1h followed by co-incubation with empagliflozin (10^−9^ or 10^−10^ M) for 48h, determined by Western blot analysis. Representative immunoblots are shown on the left, and densitometric quantification is shown on the right. Data are presented as mean ± SD from at least three independent experiments, each including a minimum of two biological replicates. Data are presented as mean ± SD. *p < 0.05, **p < 0.01, and ***p < 0.001 versus the indicated comparison group.

HIT-T15 cells were also incubated with various concentrations of tunicamycin (10 and 20 μg/ml) or empagliflozin (10^−8^ and 10^−9^ M) alone or co-incubated with both simultaneously. The incubation of HIT-T15 cells with tunicamycin resulted in a significant induction in the percentage of apoptotic and necrotic cells (*p* < 0.001) compared with untreated cells, while the co-incubation of these cells with empagliflozin (10^−8^ and 10^−9^ M) for 48h resulted in significant reduction of apoptotic cells by approximately 50% compared with cells treated with tunicamycin (10 and 20 μg/ml) alone (p < 0.05 and p < 0.01 for tunicamycin 10 μg/ml and empagliflozin 10^−8^ and 10^−9^ M, respectively, compared to tunicamycin 10 μg/ml alone; and p < 0.001 for tunicamycin 20 μg/ml and both empagliflozin 10^−8^ and 10^−9^ M compared to tunicamycin 20 μg/ml alone) (Fig. 4A).

According to these results it appears that HIT-T15 cells are more resistant to severe ER stress conditions (10 and 20 μg/ml tunicamycin) (18% and 25% of total cells were positively stained for both Annexin + PI, respectively) compared to BTC-6 cells, an effect that was significantly reversed after co-incubation of cells with empagliflozin (10^−9^ and 10^−10^ M) (reduction in the percentage of apoptotic and necrotic cells by approximately 50% and 70%, respectively). These results provide evidence that empagliflozin inhibits ER-stress induced apoptosis in both BTC-6 and HIT-T15 cell lines.

As observed in BTC-6 cells, we found that incubation of HIT-T15 cells with tunicamycin led to increased levels of CHOP compared to untreated cells *(*p < 0.001). As expected, a greater response was observed in cells incubated with the higher doses of tunicamycin (10 μg/ml and 20 μg/ml). Since we observed the beneficial effect of empagliflozin in BTC-6 cells at concentrations of 10^−9^ and 10^−10^ M, these concentrations were selected for co-incubation experiments in HIT-T15 cells. The co-incubation of cells with tunicamycin (10 μg/ml, and 20 μg/ml) and empagliflozin (10^−9^ and 10^−10^ M) led to reduced protein levels of CHOP*.* More specifically, the co-incubation of cells with both reagents resulted in a significant reduction in CHOP protein levels at all concentrations (p < 0.001) (Fig. 4B, Supplementary Fig. 1).

## 4 Discussion

T2DM is a metabolic disorder characterized by chronic hyperglycemia caused by relatively low levels of plasma insulin which are not sufficient to compensate the insulin resistance that typically co-exists and thus are unable to normalize the plasma glucose concentration [24]. The amount of insulin secreted by β-cells depends on the absolute total number of β-cells in the pancreatic islets of Langerhans (β-cell mass), as well as on the output of each β-cell (β-cell function) [3,24]. Hyperglycemia has been shown to cause β-cell toxicity and eventually β-cell death by apoptosis, through turning the balance of proapoptotic caspase family and antiapoptotic Bcl proteins towards apoptosis [25,26].

There are several molecular mechanisms underlying apoptosis of β-cells triggered by various stimuli such as islet amyloid polypeptide (IAPP) overproduction, lipotoxicity or glucotoxicity [3,[26], [27], [28], [29]]. The latter is characterized by sustained hyperglycemia and exerts deleterious and toxic effects on β-cells [3,30]. *In vitro* long-lasting exposure to elevated glucose concentrations has been shown to decrease proteasome activities leading to increased levels of polyubiquitinated proteins and finally to β-cell apoptosis through the induction of ER stress pathway [31].

According to the present findings, when BTC-6 cells (a mouse pancreatic β-cell line) are incubated with tunicamycin, which acts as an ER stress inducer, apoptosis is induced. The viability of these cells was significantly reduced after a 48h treatment with tunicamycin and the result was replicated when another β-cell line (HIT-T15) was also tested under these conditions. The mRNA expression levels of the ER stress markers *PERK*, *EIF-2α*, *CHOP* and *IRE-1α* were elevated after tunicamycin treatment, while EIF-2α protein phosphorylation and CHOP protein levels were enhanced, indicating that ER stress induction is implicated in the apoptosis of β-cells.

In line with our observations, all of the aforementioned stimuli (IAPP overproduction, lipotoxicity or glucotoxicity) that can trigger β-cell apoptosis, mediate this effect through induction of ER stress. When IAPP expression exceeds the β-cell capacity to traffic it, toxic membrane-permeant oligomers form which have been found associated with ER membranes [32], probably contributing to ER stress and apoptosis associated with it [33]. Lipotoxicity also stimulates ER stress as indicated by the induction of the expression of ER stress transducers and genes implicated in the three branches of the UPR in human islets after treatment with the fatty acid palmitate [34]. A combined transcriptomic and proteomic analysis of β-cells showed that palmitate alters genes that control ER function, ER-to-Golgi transport and ER stress pathways [35]. Triggering of ER stress which results in β-cell apoptosis has been also observed under hyperglycemic conditions. The proapoptotic transcription factor CHOP, as well as the Bcl-2 homology domain 3 (BH3)-only proteins Bim and Puma, are activated after ER stress induction and mediate cell death [36].

Furthermore, we found that empagliflozin reversed tunicamycin-induced ER stress–mediated apoptosis in BTC-6 and HIT-T15 cells. Consistent with our findings, previous studies in rodent and human islets have shown that SGLT2 is not detectably expressed in β-cells, indicating that the effects of SGLT2 inhibitors on β-cell function are unlikely to result from direct blockade of islet SGLT2. [37]. Instead, the protective actions of empagliflozin are more likely indirect and may reflect its ability to improve β-cell function and preserve β-cell mass in experimental diabetes models, with these effects commonly associated with reduced apoptosis, oxidative stress, and ER stress. In this context, empagliflozin appears to exert cytoprotective actions that extend beyond its primary renal glucose-lowering effect. Consistent with this interpretation, SGLT-2 inhibition by empagliflozin has been shown to improve β-cell function, delay pancreatic tissue damage and to restore β-cell mass in diabetic mice and rats by inhibiting inflammation and apoptosis pathways [38,39]. Cheng et al. have shown that empagliflozin administration for 8 days significantly increases the pancreatic β-cell mass in streptozotocin-induced T1D mice, while it also enhances β-cell proliferation and reduces apoptosis [17]. In the same T1D mouse model, empagliflozin treatment led to higher islet density with preserved structure, significantly less β-cell loss and total absence of immune cells infiltration [40]. The function of β-cells is also improved by empagliflozin administration in diabetic *db/db* mice in an advanced diabetes phase [18]. Similarly, luseogliflozin increased fasting insulin levels, β-cell proliferation, and the expression of Ins1, Ins2, and Glut2 in diabetic mice [41]. Consistent with these findings, dapagliflozin treatment markedly improved hyperglycemia and preserved pancreatic β-cell mass in a duration-dependent manner, with earlier administration providing greater long-term benefits [42]. Direct evidence that empagliflozin improves insulin secretion through the attenuation of ER stress remains limited [40,43]. Although one study using a tacrolimus-induced diabetes model reported increased plasma insulin levels and enhanced glucose-stimulated insulin secretion following empagliflozin treatment, *in vitro* models using isolated human and mice β-cells have not demonstrated a direct protective effect of empagliflozin on β-cell function [37,44]. In humans, β-cell function has been reported to improve rapidly after 48 h of empagliflozin treatment in patients with T2DM, with effects sustained for up to 14 days, likely as a consequence of reduced plasma glucose concentrations [45]. Similarly, another human study found that SGLT2i use was associated with lower HOMA2-IR, while some β-cell secretion indices were reduced [46], suggesting that the metabolic benefits may primarily result from improved insulin sensitivity rather than direct stimulation of insulin secretion. Altogether, these findings suggest that the beneficial effects of SGLT2 inhibitors on β-cell function are mediated mainly through reduced glucotoxicity, oxidative stress, apoptosis and ER stress, thereby preserving insulin content and glucose-stimulated insulin secretion rather than directly stimulating insulin release from isolated islets. Our investigation on the expression of *SGLT-2* at the mRNA level indicated no detection in either cell line (BTC-6 and HIT-T15). This was also observed by Chae et al. in pancreatic islets derived from mice, rats or humans, where *SGLT-*2 mRNA was absent from islet cells (α or β) and *SGLT-*1 mRNA was expressed in human β-cells, although at low levels [37]. It could be hypothesized that the effects of empagliflozin on the ER stress-induced apoptosis are mediated through SGLT-1, however, *SGLT-*1 mRNA expression was detected only in BTC-6 cells at low levels, while the antiapoptotic effect of empagliflozin through elimination of the ER stress has been confirmed also in HIT-T15 cells which express neither of the two SGLTs. Off-target and/or non-canonical effects that operate independently of SGLT-2/SGLT-1 could be a possible explanation of the favorable effects of empagliflozin on the ER-stress induced β-cell apoptosis, however, this merits further investigation. Interestingly, a recent study by Mourad et al. supported an indirect or off-target role for the cardioprotective benefits of empagliflozin in heart failure, through single cell transcriptomic analysis of SGLT2 expression [47].

According to our findings, administration of empagliflozin along with tunicamycin diminished the mRNA expression of the ER stress-implicated genes *PERK*, *EIF-2α*, *IRE-1α* and *CHOP* and significantly decreased EIF-2α protein phosphorylation in BTC-6 cells, while it also reduced CHOP protein levels in both cell lines. Daems et al. also observed an improvement in β-cell mass of T1D mice after empagliflozin treatment and examined the expression of ER stress markers in an attempt to elucidate if this improvement was due to decreased ER stress. They found that the *Xbp1* spliced form, *Txnip*, *BIP*, and *ATF-*4 mRNA levels were decreased after empagliflozin administration [40]. This is consistent with our results, indicating a role of ER stress reduction in β-cell viability preservation. However, we observed no significant changes in *BIP* and *ATF-*4 mRNA expression. Moreover, in line with our findings, the study by Shyr and colleagues noted an increased ER stress in islets of the human K_ATP_-gain-of-function induced Neonatal Diabetes Mellitus (NDM) mouse model, which was reduced after treatment with dapagliflozin, another SGLT-2i [48]. Interestingly, their results were also replicated in the leptin receptor deficient *db/db* and leptin deficient *ob/ob* mice, which serve as models of obesity and T2DM [48]. However, regarding the mRNA expression of ER stress markers, Shyr et al. identified a significant increase in *BIP* and *ATF-4* levels in NDM animals, and a significant decrease in *ATF-*4 mRNA after dapagliflozin administration, while we found no significant alterations in the expression of either of these genes. It is well known that, EIF-2 phosphorylation plays a central role in the changes of gene expression related to ER-stress induction, by enhancing the translation of ATF-4 which is a common downstream target that integrates signaling from PERK and other EIF-2 kinases. This has led to the p-EIF-2/ATF4 pathway being referred to as the integrated stress response (ISR) which either alleviates stress or triggers apoptosis [49,50]. A main ISR target gene encodes CHOP whose accumulation is critical for stress-induced apoptosis [49]. Although, we demonstrated increased PERK and EIF-2 expression, as well as increased phosphorylation of EIF-2 upon incubation with tunicamycin, as well as reversal of these effects by co-incubation with empagliflozin, the expression of *ATF-4* was not changed in either condition. Further studies are needed to investigate if other mechanisms of direct translational control of *CHOP* by the levels of phosphorylated EIF2 are involved in β-cells, as it has been shown in mouse embryonic fibroblasts (MEFs) [49].

Additionally, Shyr et al. didn't find any changes in *CHOP* mRNA levels [48] in contrast to our findings of significantly elevated *CHOP* mRNA expression in cells incubated with tunicamycin and subsequent significant reduction when these cells were co-treated with empagliflozin. These different results could be attributed, at least in part, to the different models used, i.e. *in vitro* experiment in our study vs. an animal model in the study by Shyr et al. Even in the absence of SGLT-2 and SGLT-1 expression in β-cells, SGLT-2i could reduce β-cell apoptosis indirectly through actions exerted in other tissues/organs that express SGLT-2 and/or SGLT-1 (e.g. kidney, gut, and liver) leading in lower glucotoxicity, reducing inflammation and lipotoxicity, and thus improving the islet microenvironment.

In conclusion, our study showed that *in vitro* ER stress induction by tunicamycin can lead pancreatic β-cells to apoptosis. Treatment with empagliflozin, a widely prescribed SGLT-2i, reversed this apoptotic effect by reducing ER stress, as shown by the downregulation of ER stress markers’ expression at the mRNA and protein level. Collectively, these findings suggest that SGLT-2is may protect pancreatic β-cells from death. A major limitation of this study is that we did not directly assess β-cell function, such as glucose-stimulated insulin secretion, insulin content, or β-cell mass. Therefore, although empagliflozin attenuated tunicamycin-induced ER stress and apoptosis *in vitro*, we cannot conclude that these effects necessarily translate into preserved β-cell function. In addition, while we evaluated multiple ER stress–related markers at both the mRNA (*PERK*, *eIF2α*, *CHOP*, *IRE1α*, *ATF6*, and *XBP1*) and protein (CHOP and phosphorylated eIF2α) levels, these analyses do not provide a comprehensive assessment of ER stress pathway activation. CHOP was selected as a downstream effector of the unfolded protein response; however, its expression does not distinguish the relative contribution or activation status of the individual ER stress signaling branches. Furthermore, apoptosis was assessed by Annexin V flow cytometry without evaluation of additional apoptotic markers, such as cleaved caspase-3. Future studies should therefore incorporate direct assessment of ER stress pathway activation, additional apoptotic markers, and functional β-cell analyses in both animal models and clinical settings to further define the molecular mechanisms underlying the protective effects of empagliflozin.

## Informed consent statement

Not applicable.

## Institutional review Board statement

Not applicable.

## Data availability statement

The original contributions presented in this study are included in the article. Further inquiries can be directed to the corresponding author(s).

## Funding

This research received no external funding.

## CRediT authorship contribution statement

**Narjes Nasiri-Ansari:** Formal analysis, Investigation, Methodology, Visualization, Writing – original draft. **Manpal S. Randeva:** Formal analysis, Methodology, Writing – original draft. **Christina-Maria Flessa:** Formal analysis, Investigation, Methodology, Visualization, Writing – original draft. **Ioannis Kyrou:** Project administration, Writing – review & editing. **Panagiotis Lembessis:** Methodology. **Hippokratis Kiaris:** Resources. **Maria-Eleni Chondrogianni:** Visualization, Writing – original draft. **Maria Dalamaga:** Writing – review & editing. **Athanasios G. Papavassiliou:** Conceptualization, Data curation. **Harpal S. Randeva:** Conceptualization, Data curation, Resources, Supervision, Validation, Writing – review & editing. **Eva Kassi:** Conceptualization, Data curation, Resources, Supervision, Validation, Writing – review & editing.

## Conflicts of interest

Given her role as co-Editor-in-chief, Prof Maria Dalamaga had no involvement in the peer review of this article and had no access to information regarding its peer review. Full responsibility for the editorial process regarding this article was delegated to another journal editor. The rest of the authors declare that they have no known competing financial interests or personal relationships that could have appeared to influence the work reported in this paper.

**Table:** Abbreviations

ATF-4	Activating transcription factor 4
ATF6	Activating transcription factor 6
Bcl‐2	B-Cell Leukemia/Lymphoma 2
BH3	Bcl-2 homology domain 3
BIP	Immunoglobulin heavy chain binding protein
BTC-6	Beta-TC-6 (mouse pancreatic cell line)
CHOP	C/EBP homologous protein
EIF-2α	Eukaryotic translation initiation factor 2α
Empa	Empagliflozin
ER	Endoplasmic reticulum
GADD153	Growth arrest- and DNA damage-inducible gene 153
GRP-78	78-kDa glucose-regulated protein
GRP-94	94-kDa glucose-regulated protein
HFD	High-fat diet
HIT-T15	Hamster islet cell line
IAPP	Islet amyloid polypeptide
IRE-1	Inositol-requiring kinase 1
ISR	Integrated stress response
MEFs	Mouse embryonic fibroblasts
mRNA	Messenger RNA
NDM	Neonatal Diabetes Mellitus
p-EIF-2α	Phosph-EIF2α
PERK	Protein kinase RNA-like ER kinase
SGLT-1	Sodium-glucose co-transporter 1
SGLT-2	Sodium-glucose co-transporter 2
SGLT-2i	Sodium-glucose co-transporter 2 inhibitors
T1D	Type 1 diabetes
T2DM	Type 2 diabetes mellitus
Tuna	Tunicamycin
Txnip	Thioredoxin-interacting protein
UPR	Unfolded protein response
Xbp1	X-box binding protein 1

## Appendix A Supplementary data

Fig. S1 Original western blot films in accordance to their appearance in Fig. 3, Fig. 4B.
