# Supplementary material for: Empagliflozin inhibits the apoptosis of pancreatic β-cells through amelioration of endoplasmic reticulum (ER) stress
Source: Metabol Open. 2026 Aug 2;31:100488. doi: 10.1016/j.metop.2026.100488 (PMC13470530; doi:10.1016/j.metop.2026.100488)
Supplement: Supplementary file 1 [file mmc1.pdf]

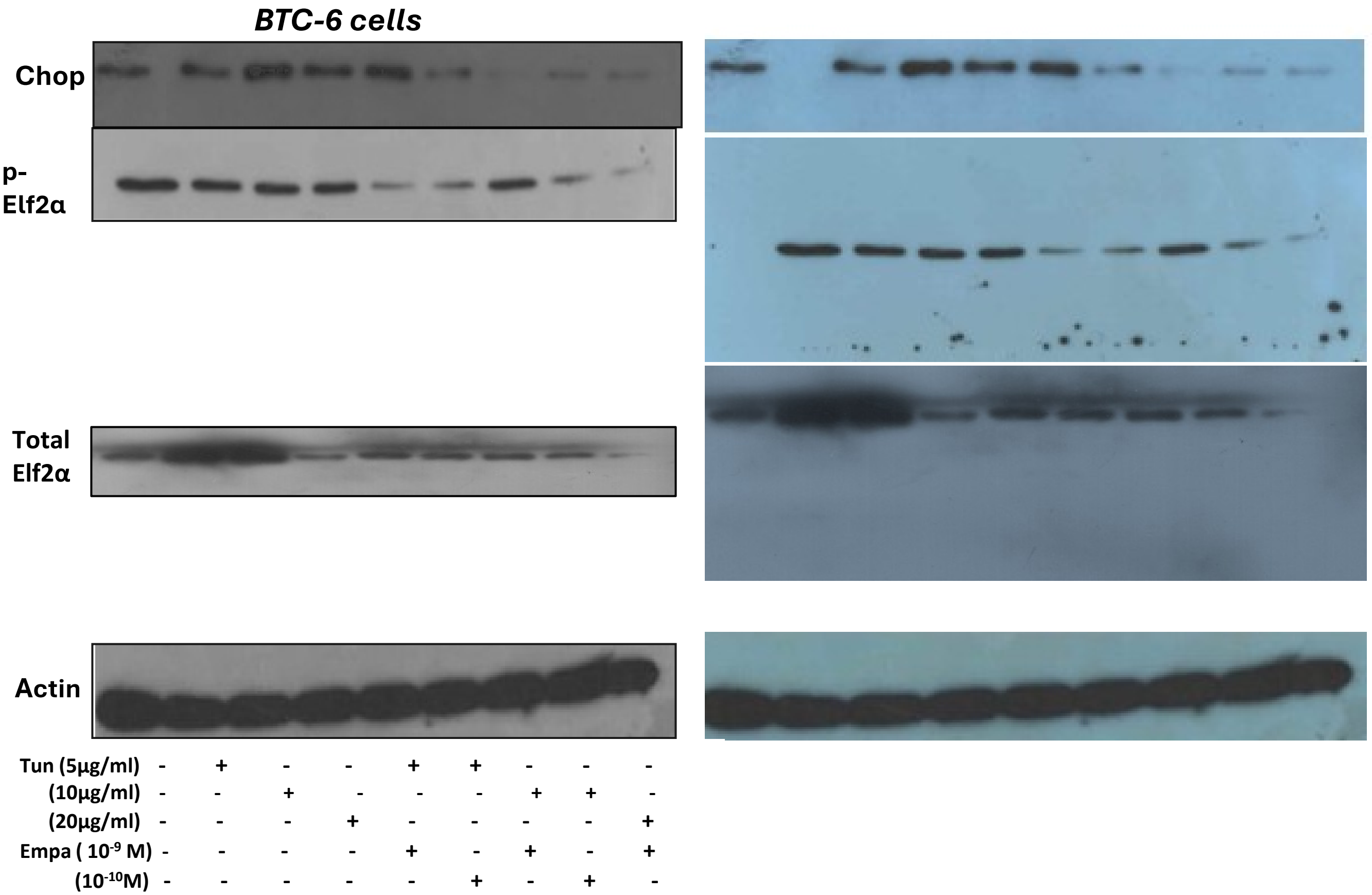

Figure 3.

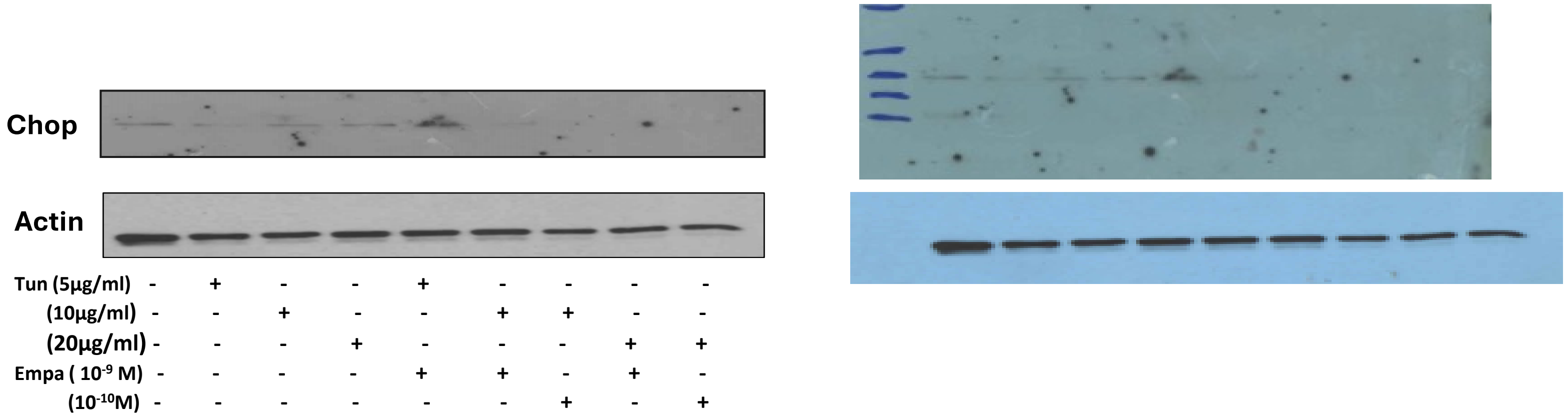

Figure 4.B
